# Associations between abstract working memory abilities and brain activity underlying long-term recognition of auditory sequences

**DOI:** 10.1093/pnasnexus/pgac216

**Published:** 2022-09-28

**Authors:** Gemma Fernández-Rubio, Francesco Carlomagno, Peter Vuust, Morten L Kringelbach, Leonardo Bonetti

**Affiliations:** Center for Music in the Brain, Department of Clinical Medicine, Aarhus University & The Royal Academy of Music Aarhus/Aalborg, 8000 Aarhus, Denmark; Center for Music in the Brain, Department of Clinical Medicine, Aarhus University & The Royal Academy of Music Aarhus/Aalborg, 8000 Aarhus, Denmark; Department of Education, Psychology, Communication, University of Bari Aldo Moro, 70121 Bari BA, Italy; Center for Music in the Brain, Department of Clinical Medicine, Aarhus University & The Royal Academy of Music Aarhus/Aalborg, 8000 Aarhus, Denmark; Center for Music in the Brain, Department of Clinical Medicine, Aarhus University & The Royal Academy of Music Aarhus/Aalborg, 8000 Aarhus, Denmark; Centre for Eudaimonia and Human Flourishing, Linacre College, University of Oxford, Oxford OX3 9BX, UK; Department of Psychiatry, University of Oxford, Oxford OX1 2JD, UK; Center for Music in the Brain, Department of Clinical Medicine, Aarhus University & The Royal Academy of Music Aarhus/Aalborg, 8000 Aarhus, Denmark; Centre for Eudaimonia and Human Flourishing, Linacre College, University of Oxford, Oxford OX3 9BX, UK; Department of Psychiatry, University of Oxford, Oxford OX1 2JD, UK

**Keywords:** recognition memory, working memory, brain activity, predictive coding of music (PCM), magnetoencephalography (MEG)

## Abstract

Memory is a complex cognitive process composed of several subsystems, namely short- and long-term memory and working memory (WM). Previous research has shown that adequate interaction between subsystems is crucial for successful memory processes such as encoding, storage, and manipulation of information. However, few studies have investigated the relationship between different subsystems at the behavioral and neural levels. Thus, here we assessed the relationship between individual WM abilities and brain activity underlying the recognition of previously memorized auditory sequences. First, recognition of previously memorized versus novel auditory sequences was associated with a widespread network of brain areas comprising the cingulate gyrus, hippocampus, insula, inferior temporal cortex, frontal operculum, and orbitofrontal cortex. Second, we observed positive correlations between brain activity underlying auditory sequence recognition and WM. We showed a sustained positive correlation in the medial cingulate gyrus, a brain area that was widely involved in the auditory sequence recognition. Remarkably, we also observed positive correlations in the inferior temporal, temporal-fusiform, and postcentral gyri, brain areas that were not strongly associated with auditory sequence recognition. In conclusion, we discovered positive correlations between WM abilities and brain activity underlying long-term recognition of auditory sequences, providing new evidence on the relationship between memory subsystems. Furthermore, we showed that high WM performers recruited a larger brain network including areas associated with visual processing (i.e., inferior temporal, temporal-fusiform, and postcentral gyri) for successful auditory memory recognition.

Significance Statement Memory is dependent on the successful interaction between its multiple subsystems. Here, we assessed the relationship between individual working memory (WM) abilities and brain activity underlying the recognition of previously memorized auditory sequences. We observed positive correlations between brain activity underlying auditory sequence recognition and WM, especially in the medial cingulate gyrus, inferior temporal, temporal-fusiform, and postcentral gyri. In this study, we provided new evidence on the relationship between two memory subsystems: WM and long-term auditory recognition. Moreover, we showed that, to successfully complete memory recognition tasks, high WM performers recruited a larger brain network, which comprised brain areas mainly associated with visual processing, such as inferior temporal, temporal-fusiform, and postcentral gyri.

## Introduction

Memory is a fundamental cognitive process that is widely regarded as a multisystem function (1) relying on a widespread network of brain areas such as the medial temporal lobe (2, 3), prefrontal cortex (4), and basal ganglia (5). Broadly, the memory subsystems encode, store, and retrieve past memories (long-term memory), temporarily store sensory information (short-term memory), and maintain and manipulate data [working memory (WM)] (1, 6, 7). These subsystems operate simultaneously and in parallel (8), giving rise to efficient memory functioning that is essential for many daily activities.

WM capacity allows us to briefly store and manipulate information and is involved in decision-making and executive processes (9–11). Among the several theories of WM, Baddeley and Hitch's (12) multicomponent model has become highly influential. According to this theory and its subsequent revisions, WM is composed of four components: (i) the phonological loop, which is involved in verbal WM; (ii) the visuospatial sketchpad, for visuospatial WM; (iii) the central executive, or the attentional control system; and (iv) the episodic buffer, for storing information (10, 12–14). Frequently, WM paradigms request individuals to retain sensory information and perform some operation or manipulation on it, as in the case of the *N*-back (15) and digit span (16) tasks.

Neuroimaging studies have highlighted the role of cortical brain areas, such as the prefrontal, parietal, and cingulate cortices, and subcortical areas, including the midbrain and cerebellum, in WM processes, as reported in a review by Chai et al. (17). Evidence comes mainly from studies using visual stimuli, providing a valuable but incomplete picture of the neuroanatomy of WM. However, recent studies on auditory WM processing have uncovered the role of the primary auditory cortex and high-order structures such as the hippocampus for this cognitive function. For example, Kumar and colleagues (18) demonstrated that the activity and connectivity of the primary auditory cortex, hippocampus, and inferior frontal gyrus are associated with the maintenance of single sounds’ series. Additionally, theta oscillations and phase locking in the dorsal stream predict performance in a maintenance and manipulation auditory task (19). Related to the present study, Bonetti et al. (20) showed a positive correlation between WM capacity and brain activity underlying an auditory mismatch-negativity (MMN) task. The authors found that participants with higher WM scores showed enhanced MMN responses in frontal regions but not in temporal areas. Notably, this investigation evidenced the relationship between auditory short-term memory and WM.

Long-term memory refers to the ability to recall information that has been encoded and stored in the past (7, 21). Research on this cognitive function has emphasized the distinct features of several types of long-term memory, namely episodic, semantic, and procedural memory (22, 23). These are classified according to the kind of information they hold (e.g. personal experiences in the case of episodic memory, factual knowledge for semantic memory) (24, 25) and how this information is encoded (e.g. skill acquisition in procedural memory) (26).

The neural underpinnings of long-term memory rest primarily upon medial temporal lobe structures (hippocampus, entorhinal, perihinal, and parahippocampal cortices) (2, 21) and interact with the prefrontal cortex for successful memory retrieval (27). Moreover, consolidation, the process of transforming temporary information into long-lasting memories, is achieved through the interactions between the hippocampus and neocortex (28, 29). Converging evidence suggests that, in the case of auditory long-term memory, the primary auditory cortex also supports the storage of information (30). Using a musical long-term memory task, Groussard et al. (31) revealed that musicians’ inferior frontal and superior temporal gyri, inferior parietal gyrus, cerebellum, and hippocampus are active when judging the level of familiarity of musical excerpts. Additionally, similar to visual long-term memory, recognition of previously memorized environmental sounds elicits stronger gamma-band activity in central electrodes than listening to novel sounds (32).

Although previous investigations have mainly examined the neuroanatomical bases of the memory subsystems in isolation, few studies have looked into the associations between them. For instance, Henson and Gagnepain (33) highlighted the interaction between different memory subsystems, both in terms of behavior and neural substrate. They focused especially on episodic, semantic, and modality-specific perceptual subsystems, claiming that their successful interaction is crucial for performing memory tasks. Similarly, Poldrack and colleagues (34) demonstrated the interaction and competition between memory subsystems during classification learning in humans. Specifically, they observed that the basal ganglia and medial temporal lobe were differently engaged depending on the emphasis on declarative or nondeclarative memory and showed that the interaction between these structures was necessary to perform the task. In a review focusing on pharmacological and neurochemical studies, Gold (35) proposed that the release of acetylcholine in different memory subsystems showed extensive interactions between them, which could be cooperative or competitive. He concluded that different memory and neural systems tended to interact extensively, even when described as relatively independent. Finally, White and McDonald (36) described a theory of multiple parallel memory subsystems in the rat brain localized in the hippocampus, caudate-putamen, and amygdala. The authors claimed that all subsystems had access to the same information during learning, but that each subsystem represented a different relationship between the information features. In their view, these memory subsystems interacted by simultaneous parallel influence on behavioral output and by directly affecting each other in a cooperative or competitive manner. Overall, these investigations have yielded considerable insights into the relationships between memory subsystems, but we still lack information on the brain correlates underlying these interactions.

Thus, in our study, we aimed to investigate the relationship between two of the most important memory subsystems, WM and long-term memory, emphasizing their interdependence. To this end, we correlated the scores from a widely used auditory WM measure with the neural activity underlying tone-by-tone recognition of previously memorized sequences from three different musical pieces. Previously, we found that WM abilities have a minor but significant impact on the brain encoding processes of a musical piece (37). Following this result, we hypothesized to observe stronger brain activity underlying auditory sequence recognition in individuals with greater WM abilities, especially in brain structures that have been previously associated with memory processes, such as the prefrontal cortex and hippocampus (37). Additionally, we expected WM capacity to be positively correlated with behavioral responses in the auditory recognition task.

## Results

### Experimental design

**Fig. 1.**
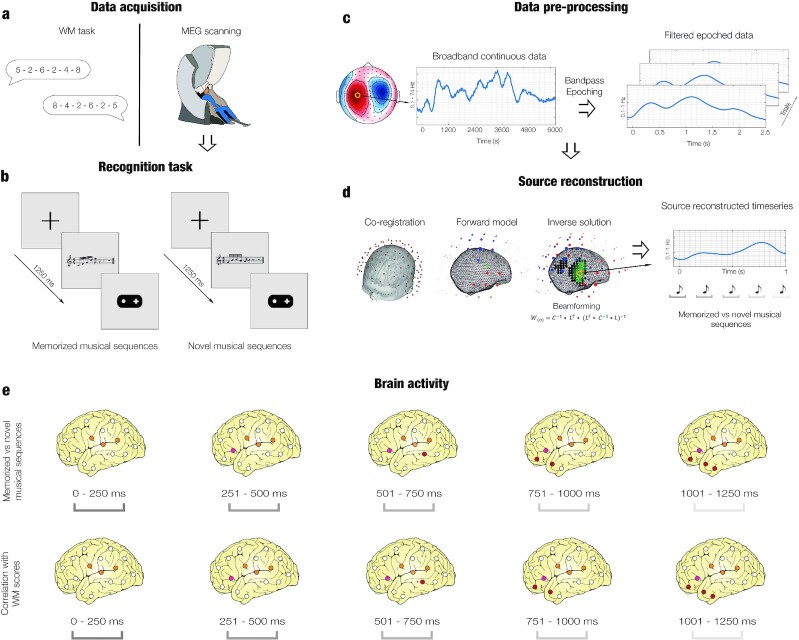
Experimental stimuli and design, and data analysis overview. (a) The data acquisition comprised two parts: (i) a WM task completed outside the scanner and (ii) an old/new auditory recognition task that was carried out during MEG recording. (b) Illustration of the old/new auditory recognition task performed in the MEG scanner. After listening to three full musical pieces, participants were presented with melodic excerpts that were extracted from the pieces they previously learned or with new melodies, and they were asked to state whether each melody was memorized or novel using a joystick. (c) The broadband continuous neural data was preprocessed, bandpass filtered (0.1 to 1 Hz),  and epoched. (d) Source reconstruction analyses were performed to isolate the contribution of each brain source to the neural activity recorded by the MEG sensors. Preprocessed MEG and MRI data were coregistered. After that, a forward model was computed, and the inverse solution was estimated using a beamforming approach. (e) Contrasts between memorized and novel auditory sequences were calculated for each musical tone (top row). Pearsons’ correlations between WM scores and brain activity underlying recognition of memorized versus novel auditory sequences were computed (bottom row).

Participants performed an old/new auditory recognition task (38–40). During the encoding phase, participants listened to three musical pieces and were instructed to memorize them as much as possible. In the recognition phase, short musical sequences selected from the pieces (i.e. memorized musical sequences) and novel musical sequences were presented. For each of the sequences, participants stated whether they were memorized or novel. Due to its high temporal resolution, their brain activity was recorded using magnetoencephalography (MEG) during the recognition task. Structural magnetic resonance imaging (MRI) images were collected for each participant and combined with the MEG data to reconstruct the sources using a beamforming approach, which generated the signal that was recorded over the MEG channels. Finally, participants’ WM abilities were measured using the Digit Span and Arithmetic subtests from the Wechsler Adult Intelligence Scale (WAIS-IV) (41). Fig. 1 shows a graphical depiction of the experimental design and analysis pipeline.

### Brain activity underlying recognition of previously memorized versus novel musical sequences

Before evaluating the relationship between WM abilities and brain activity underlying musical sequence recognition, which was the main aim of the current work, we wished to replicate the established finding (38–40) that recognition of previously memorized versus novel auditory sequences is associated with a stronger activation in a widespread network of brain areas. Since previous studies showed that the slow frequency band of 0.1 to 1 Hz was crucially associated with the recognition of the complete musical sequence, we performed this analysis at the same slow frequency band (38–40).

First, we subaveraged the brain data in five time-windows, corresponding to the duration of the five tones of the musical sequences (0 to 250 , 251 to 500, 501 to 750, 751 to 1000, and 1001 to 1250 ms). Second, independently for the five time-windows, we computed one t-test for each brain source, contrasting the brain activity underlying recognition of previously memorized versus novel musical sequences. Third, we corrected for multiple comparisons using cluster-based Monte Carlo simulations (MCS).

Significant clusters of activity (*P* < .001) were located across a number of brain voxels (*k*) for each tone of the musical sequences. As expected, the main clusters were observed for the third (*k* = 284), fourth (*k* = 390), and fifth tones (*k* = 125). The strongest differences between the two conditions were localized in the middle cingulate gyrus, precuneus, insula, hippocampal regions, orbitofrontal cortex, and frontal operculum.

**Fig. 2.**
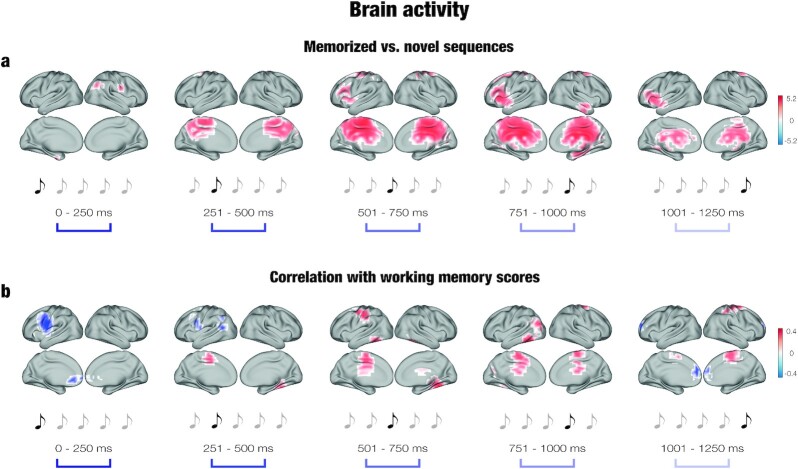
Brain activity underlying the recognition of auditory sequences and correlation with WM scores. (a) Significant brain activity underlying recognition of the musical sequences. The activity is depicted in brain templates in five subsequent time windows corresponding to the duration of each musical tone forming the sequences (as illustrated by the sketched musical tones above the time windows). The colorbar shows the *t*-values resulting from the contrast between memorized and novel auditory sequences. (b) Significant Pearson's correlations between the brain activity underlying recognition of the sequences and WM scores. The correlations are depicted in brain templates in five subsequent time windows corresponding to the duration of each musical tone forming the sequences (as illustrated by the sketched musical tones above the time windows). The colorbar shows Pearson's correlation coefficient obtained by correlating the brain activity underlying recognition of the previously memorized versus novel auditory sequences with the WM scores.

Detailed statistics and information for each voxel forming the significant clusters are reported in supplementary materials (Table S1), while a graphical depiction of the results is illustrated in Fig.2a.

### WM abilities and brain activity underlying musical sequence recognition

The main aim of the study was to establish whether there was a significant relationship between WM abilities and brain activity underlying tone-by-tone recognition of musical sequences.

Before computing neural data analyses, we calculated a Pearson's correlation between the number of correctly recognized auditory sequences in the MEG task and the individual WM scores. The analysis returned a nonsignificant result (*rho* = .16, *P* = .18).

To address our experimental question, we computed Pearson's correlations between participants’ WM scores and each of the reconstructed brain sources. We corrected for multiple comparison using cluster-based MCS. This procedure was computed independently for five time-windows, corresponding to the duration of the five tones of the musical sequences (0 to 250, 251 to 500, 501 to 750, 751 to 1000, and 1001 to 1250 ms; see the ‘‘Methods” section for details).

Significant clusters of activity (*P* < .05) were located in different brain regions and depicted an overall positive correlation between WM abilities and brain activity underlying recognition of memorized musical sequences. This difference returned consistent clusters in the middle cingulate gyrus, inferior temporal cortex, fusiform-temporal cortex, parahippocampal gyrus, and temporal-occipital fusiform cortex, especially for the third (*k* = 83) and fourth (*k* = 83) tones of the musical sequences.

Detailed statistics and information for each voxel forming the significant clusters are reported in the Supplementary Materials (Table S2), while a graphical depiction of the results is illustrated in Fig. 2b.

Additional analyses were conducted to examine the impact of musical training on the relationship between WM abilities and auditory memory recognition, based on previous studies linking musical training with improved auditory memory (42, 43). We used the Goldsmiths Musical Sophistication Index (Gold–MSI) (44) to measure musical expertise, and the same procedure was repeated for two groups of participants (musicians and nonmusicians) independently. Significant clusters of activity (*P* < .025) in right hippocampal areas showed a positive correlation between WM abilities and memory recognition for both musicians and nonmusicians. Additionally, activity in the medial cingulate gyrus was positively correlated with WM scores in the case of musicians only, while activity in the left auditory cortex was correlated with WM scores in nonmusicians. Detailed statistics and information are reported in the Supplementary Materials (Fig. S1 and Tables S3 and S4).

## Discussion

In this study, we assessed the relationship between individual WM abilities and brain activity underlying long-term recognition of auditory sequences.

First, we identified the brain activity associated with recognition of previously memorized versus novel auditory sequences. This analysis revealed a widespread network of brain areas involved in the recognition process, including the cingulate gyrus, hippocampus, insula, inferior temporal cortex, frontal operculum, and orbitofrontal cortex. Remarkably, the cingulate gyrus (especially the posterior part) was significantly more active for memorized than for novel sequences by the second tone of the sequence. Moreover, this region was strongly active during the processing of the rest of the sequence, although its activity decreased in the last tone. Conversely, the insula, inferior temporal cortex, and hippocampal areas were mainly active during the third, fourth and fifth tones of the auditory sequence.

Second, we correlated the brain activity underlying recognition of memorized versus novel sequences with the participants’ WM scores. In general, we observed positive correlations between brain activity and WM capacity. The analyses returned a sustained positive correlation in the medial cingulate gyrus, a brain region strongly involved in auditory sequence recognition. Notably, we also observed positive correlations in the inferior temporal, temporal-fusiform, and postcentral gyri. These brain areas were not strongly associated with auditory sequence recognition and suggest that high WM performers may recruit a larger brain network to successfully complete memory recognition tasks. Remarkably, despite the positive correlation between WM capacity and brain activity, scores on the WM task and the recognition task were not correlated. Although unexpected, this result points to the relevance of the brain activity correlation to provide crucial information that the behavioral data could not return.

Our results on the whole-brain mechanisms for auditory recognition are coherent with previous studies that employed the same paradigm. For instance, using part of the current dataset, Bonetti et al. (38, 39) and Fernández-Rubio et al. (40) highlighted the crucial role of the cingulate gyrus, hippocampus, insula, inferior temporal cortex, and frontal operculum for the recognition of auditory sequences. Similarly, in another study, Bonetti et al. (37) found that the degree centrality of the frontal operculum within the whole brain network during the encoding of a musical piece was positively correlated with WM scores. The replication of previous findings encouraged us to further investigate the relationship between brain activity underlying auditory sequence recognition and individual WM skills.

Overall, this study showed a series of positive correlations between brain activity and WM abilities, suggesting that memory subsystems are coherently connected to each other. This is particularly interesting since the recognition task employed in the study used musical stimuli, while the WM measure was based on numbers. This link between different subsystems of memory is in line with previous research. As previously mentioned, the nature of the interactions between subsystems may be cooperative or competitive (34, 35) and is essential to perform memory tasks efficiently (33). Furthermore, different brain areas are involved depending on the memory process that is emphasized (declarative versus nondeclarative) (34). Finally, White and McDonald's (36) study localized multiple parallel memory subsystems in the rat's hippocampus, caudate-putamen, and amygdala, and proposed that these subsystems share information during learning but represent its features differently.

Of particular interest in this study are the brain areas that were connected to WM. The activity recorded in the medial cingulate gyrus presented a sustained positive correlation with WM scores. This is coherent with previous studies linking cingulate gyrus’ activity to memory and musical tasks. As mentioned earlier, in the auditory domain, the cingulate played a crucial role in auditory sequence encoding (37) and recognition (38–40). Moreover, a recent meta-analysis revealed that the cingulate gyrus is central for general music processing and particularly for sound imagination (45). Beyond the auditory system, the cingulate gyrus has been reported in memory studies employing visual or abstract information. For instance, it has been suggested that diverse parts of the cingulate gyrus are differently involved in memory processes. According to this view, the anterior part of the cingulate is primarily connected to the orbitofrontal cortex and handles abstract reward outcomes, while the posterior cingulate is integrated within the hippocampal and occipital systems and therefore highly relevant for memory processing of visual stimuli (46, 47). Similarly, in a recent functional magnetic resonance imaging (fMRI) study, Di and colleagues (48) showed that the anterior cingulate gyrus was functionally connected to the middle frontal gyrus and superior parietal lobule during a demanding WM task. Conversely, this connectivity was reduced in the resting state, suggesting the relevance of the cingulate gyrus during memory tasks.

Other brain structures correlated with WM abilities were the inferior temporal and temporal-fusiform gyri and the postcentral gyrus. This result is of great interest because these brain structures did not play a major role in the recognition of auditory sequences. Indeed, while the cingulate gyrus was largely active, we previously observed a relatively small contribution of inferior temporal and postcentral gyri to auditory sequence recognition (38–40). Moreover, the temporal-fusiform gyrus has not been previously associated with auditory recognition processes. This suggests that individuals with higher WM abilities recruited a larger brain network during the recognition of auditory sequences, which may provide an advantage for auditory recognition. However, since there were no significant differences in the behavioral performance of the recognition task, future studies are called to better understand whether and how this recruitment of additional brain areas is beneficial for individuals with high WM capacity.

Previous literature has shown the involvement of inferior temporal and temporal-fusiform gyri and the postcentral gyrus in visual memory tasks. In the past decades, the inferior temporal cortex has been widely connected to visual perception and memory in both humans and monkey (49). Specifically, several studies demonstrated the involvement of the inferior temporal cortex in representational memory and recognition of complex visual patterns (49, 50). More recently, Costers and colleagues (51) reported the involvement of left and right inferior temporal and parahippocampal gyri in a multi-item WM task. Activity in the inferior temporal gyrus has been repeatedly observed in visual memory tasks, while its involvement in the auditory domain is less established. Importantly, here we revealed that the inferior temporal cortex plays a significant role in auditory recognition, at least in individuals with superior WM skills.

The fusiform gyrus has been historically connected to recognition in the visual domain, especially in relation to faces (52–55). However, recent studies demonstrated its involvement in the recognition and processing of more general visual stimuli, such as letters (56, and when performing elaborated associative learning tasks (57).

The postcentral gyrus is a brain area mainly associated with motor control(58, 59), yet evidence points to its contribution to memory processes. For instance, in a visual encoding task, a vast network of brain areas was active, including the postcentral gyrus (60). Similarly, in a recognition task of short sentences, supramarginal and postcentral gyrus activity was reported (61). Another study demonstrated the involvement of the postcentral gyrus in a WM and especially in a visual attention task (62). Notably, similar to the inferior temporal gyrus, previous literature reported activation of the postcentral gyrus mainly in relation to visual memory (60, 61), while this study showed its involvement during recognition of auditory temporal sequences. Since in this study all participants provided motor responses, future studies are called to examine the distinct contributions of the postcentral gyrus to memory processes and motor control.

Finally, supplementary analyses returned significant results when examining the correlation between WM abilities and the brain activity underlying auditory long-term memory recognition in two groups of musicians and nonmusicians. We found that hippocampal regions and the inferior temporal cortex in the right hemisphere were crucially associated with WM scores in both groups. Conversely, the medial cingulate gyrus was mainly relevant for participants with previous musical training. This last result is coherent with our recent meta-analysis that showed the key involvement of the medial cingulate gyrus during musical imagery and performance (45). Activity in the left auditory cortex was correlated with WM scores, mainly in nonmusicians. However, since the current study was not specifically designed to test differences between musicians and nonmusicians in this relationship, future studies are called to investigate this with a larger sample size and carefully controlling musical training level.

In conclusion, we discovered a positive correlation between individual WM abilities and brain activity underlying recognition of memorized auditory sequences, increasing our knowledge on the relationships between different memory subsystems. Although the methodology employed in this study is well-established and neuroimaging data were collected from 70 participants, we acknowledge that a larger sample size would highlight even better the individual differences in WM abilities and auditory long-term memory. Additionally, despite the high temporal resolution of MEG, it would be advantageous to examine the spatiotemporal dynamics of auditory long-term memory using fMRI to confirm and expand our results regarding the neural correlates of this process. Finally, future studies are encouraged to replicate our results and those of Bonetti et al. (37) and expand them by investigating the relationship between the brain mechanisms underlying long-term encoding and recognition of temporal sequences and the brain processes associated with WM tasks.

## Materials and Methods

### Participants

We recruited 71 participants (38 males and 33 females) who took part in the experiment on a voluntary basis. They were aged 18 to 42 y old (mean age: 25 ± 4.10 y). All participants were healthy and had normal hearing. Participants came from Western countries and had homogenous educational and socioeconomic backgrounds. Before starting the experimental procedures, participants gave their informed consent and performed a hearing test to adjust the volume level individually.

This study was a part of a larger project focused on brain dynamics underlying encoding and recognition of musical patterns. This project produced several studies (37–40). In the current work, we used the brain activity data underlying recognition of musical patterns that was previously reported in Bonetti et al. (38,39) and Fernández-Rubio et al. (40). The project was approved by the Ethics Committee of the Central Denmark Region (De Videnskabsetiske Komitéer for Region Midtjylland, (Ref 1-10-72-411- 17). Moreover, the experimental procedures complied with the Declaration of Helsinki Ethical Principles for Medical Research.

### Experimental stimuli and design

The study aimed at investigating the relationship between brain activity during a memory recognition task and WM abilities (Fig. 1).

The brain activity was measured using MEG, while participants performed an old/new auditory recognition task. The task consisted of an encoding phase during which participants memorized a musical piece, and a recognition phase in which they recognized excerpts from the piece. In the encoding phase, participants were exposed to four repetitions of a full musical piece and were asked to memorize it as much as they could. The musical piece lasted for approximately 2.5 minutes. The total duration of the learning phase was approximately 10 minutes. For the recognition phase, 40 short excerpts (5-tone musical sequences, 1250 ms of duration in total) were extracted from the musical piece and 40 novel musical sequences were created. The resulting 80 sequences were presented in a randomized order. For each of them, participants were instructed to state whether the sequence was extracted from the musical piece they previously learned (memorized sequence) or whether it was a new sequence (novel sequence). To prevent from potential confounds, memorized and novel sequences were matched among several variables, including rhythm, timbre, volume, meter, tempo, number, duration of musical tones, tonality, information content (*IC*), and entropy (*H*).

This task was conducted independently for three musical pieces composed in different musical tonalities, with the aim of collecting a copious amount of data and increase the reliability of our findings. The three musical pieces were the right-hand part of J. S. Bach's Prelude No. 1 in C minor BWV 847 the right-hand part of J. S. Bach's Prelude No. 1 in C major BWV 846 and an atonal version of the “major prelude” All the pieces had the same duration. The atonal piece was composed by LB following a systematic change of pitch of the tones of the major prelude. Additional details on this procedure can be found in Fernández-Rubio et al. (40).

The MIDI versions of the three pieces used in the encoding phase and the musical sequences used in the recognition phase were created using using Finale (MakeMusic, Boulder, CO, USA) and presented to the participants through Presentation software (Neurobehavioural Systems, Berkeley, CA, USA).

WM abilities were assessed with the Wechsler Adult Intelligence Scale IV (WAIS-IV) (41), one of the most widely used tests to assess cognitive abilities. The WAIS-IV comprises four main indices: WM, Verbal Comprehension, Perceptual Reasoning, and Processing Speed. In this study, we used the two primary subtests of the WM index: Digit Span and Arithmetic. In the Digit Span subtest, participants are presented orally with sequences of numbers and required to repeat them in the same order, backwards, or in ascending order, immediately after hearing them. In the Arithmetic subtest, participants are presented orally with mathematical problems and are required to solve them without using any external aids (e.g. calculator, pen, etc.). These tests were performed outside the scanner.

### Data acquisition

The MEG data were recorded in a magnetically shielded room located at Aarhus University Hospital (Denmark) with an Elekta Neuromag TRIUX MEG scanner equipped with 306 channels (Elekta Neuromag, Helsinki, Finland). The data were collected at a sampling rate of 1000 Hz with an analogue filtering of 0.1 to 330 Hz. Before starting the experiment, we recorded the participants’ headshape and the position of four Head Position Indicator (HPI) coils with respect to three anatomical landmarks (nasion and left and right preauricular points) using a 3D digitizer (Polhemus Fastrak, Colchester, VT, USA). We used this information in a later stage of the analysis pipeline to coregister the MEG data with the MRI anatomical images. During the MEG experiment, the HPI coils recorded the continuous head localization, which was subsequently used to compensate for participants’ movement inside the MEG scanner. Moreover, two sets of bipolar electrodes were employed to record cardiac rhythm and eye movements. These were later used to remove electrooculography (EOG) and electrocardiography (ECG) artifacts.

The MRI scans were acquired on a CE-approved 3T Siemens MR-scanner at Aarhus University Hospital (Denmark). We recorded a structural T1 with a spatial resolution of 1.0 × 1.0 × 1.0 mm and the following sequence parameters: echo time (TE) = 2.96 ms, repetition time (TR) = 5000 ms, and bandwidth = 240 Hz/pixel, and reconstructed matrix size = 256 × 256.

The MEG and MRI recordings were acquired in two separate sessions.

### Data preprocessing

The raw MEG sensor data (204 planar gradiometers and 102 magnetometers) was preprocessed by MaxFilter (63) in order to suppress external artifacts interfering with the magnetic field produced by the brain activity. Using MaxFilter, the data were also corrected for head motion and downsampled to 250 Hz. We then converted the data into Statistical Parametric Mapping (SPM) (64) format and further analyzed it in MATLAB (MathWorks, Natick, MA) using the Oxford Centre for Human Brain Activity (OHBA) Software Library (OSL, https://ohba-analysis.github.io/osl-docs/), a freely available software that builds upon Fieldtrip (65), FSL (66), and SPM toolboxes, and in-house-built functions. We applied a notch filter to the data (48 to 52 Hz) to correct for inferences of the electric current. The signal was further downsampled to 150 Hz and the continuous MEG data were visually inspected to control for artifacts. To remove EOG and ECG components, we computed independent component analyses (ICA), isolated and discarded the components that picked up the EOG and ECG activity, and reconstructed the signal with the remaining components. We then bandpass-filtered the data in the 0.1 to 1 Hz band, since we had previously shown (38–40) that activity in this slow frequency is mainly associated with the recognition of musical sequences. The data were subsequently epoched into 80 trials (40 memorized and 40 novel musical sequences), independently for the recognition of the three musical preludes. Then, we merged the three datasets, obtaining 240 trials (120 memorized and 120 novel musical sequences) without differentiating between the three musical preludes. Here, each trial lasted 3500 ms (3400 ms plus 100 ms of baseline time) and further analyses were performed on correctly identified trials only.

### Source reconstruction

After computing the preprocessing of the data, we estimated the brain sources that generated the signal recorded by the MEG. This procedure was carried out by designing a forward model and computing the inverse solution using beamforming algorithms .   Fig. 1 shows an illustration of the source reconstruction pipeline.

First, using the information collected with the 3D digitizer, the MEG data and the individual T1-weighted images were coregistered, independently for each participant. We used the MNI152-T1 standard template with 8-mm spatial resolution in the case of four participants whose individual anatomical scans were not available.

Second, we computed a single-shell forward model using an 8-mm grid. This theoretical head model considers each brain source as an active dipole and calculates how a unitary strength of such dipoles would be reflected over the MEG sensors (67). Then, we used a beamforming algorithm as inverse model. This is one of the most used algorithms for reconstructing the brain sources from MEG channels’ data. It consists of employing a different set of weights based on the forward model and the covariance between the MEG channels. Afterwards, these weights are sequentially applied to the source locations (dipoles) for computing the contribution of each source to the activity recorded by the MEG channels, independently for each time point(68–70).

### Brain activity underlying recognition of previously memorized versus novel musical sequences

Before evaluating the relationship between WM abilities and brain activity underlying musical sequence recognition, which was the main aim of the current work, we wished to replicate the established finding (38–40) that recognition of previously memorized versus novel auditory sequences is associated with a stronger activation of a widespread network of brain areas. This analysis was performed at a slow frequency (0.1 to 1 Hz) that has been linked to memory recognition processes in previous studies [see Bonetti et al. (38, 39) and Fernández-Rubio et al. (40) for details].

Thus, we first subaveraged the brain data in five time-windows corresponding to the duration of the five tones of the musical sequences (0 to 250, 251 to 500, 501 to 750, 751 to 1000, and 1001 to 1250 ms). Second, independently for the five time-windows, we computed one t-test for each brain source, contrasting the brain activity underlying recognition of previously memorized versus novel musical sequences. Third, we corrected for multiple comparisons using cluster-based MCS.

Cluster-based MCS returned the spatial clusters of brain sources that exhibited a significantly different activity between our two experimental conditions (*α* = .001). Then, the significant brain voxels emerged from the previous t-tests were shuffled in space and the maximum cluster size was measured. Repeating this procedure for each of the 1,000 permutations used in the MCS analysis, we built a reference distribution of the maximum cluster sizes computed in the permuted data. Then, the original cluster sizes were compared to the reference distribution and were considered significant only if their size was bigger than the 95% of the maximum cluster sizes of the permuted data.

### WM abilities and brain activity underlying recognition of musical sequences

Before computing neural data analysis, we inspected whether there was a relationship between recognition accuracy and WM skills. To this aim, we computed a Pearson's correlation between the individual WM scores (from WAIS-IV) and the number of correctly recognized auditory sequences in the MEG task.

To determine the relationship between WM abilities and brain activity underlying recognition of musical sequences, we computed Pearson's correlations between participants’ WM scores and each of the reconstructed brain sources. We corrected for multiple comparisons using cluster-based MCS analogous to the ones described in the previous subsection. This procedure was computed independently for five-time windows that corresponded to the duration of the five tones of the musical sequences (0 to 250, 251 to 500, 501 to 750, 751 to 1000, and 1001 to 1250 ms). Cluster-based MCS returned the spatial clusters of active brain sources during recognition of musical sequences that significantly correlated (*α* = .05) with the participants’ WM abilities. For each of the five MCS, the data was subaveraged in the correspondent time window (as reported above), and the brain activity underlying recognition of novel sequences was subtracted from the brain activity underlying recognition of memorized sequences. In this way, we correlated the WM scores with the brain activity that was associated with the recognition of the sole memorized sequences. Then, the significant brain voxels emerged from the previous correlations were shuffled in space and the maximum cluster size was measured. Repeating this procedure for each of the 1,000 permutations used in the MCS analysis, we built a reference distribution of the maximum cluster sizes computed in the permuted data. Finally the original cluster sizes were compared to the reference distribution and were considered significant only if their size was bigger than the 95% of the maximum cluster sizes of the permuted data.

Following previous works on the positive impact of musical training on auditory memory (42, 43), additional analyses were conducted to investigate the relationship between WM abilities and the brain activity underlying auditory long-term memory recognition in musicians and nonmusicians. Participants’ formal musical training was measured with the Musical Training facet of Gold-MSI (44). The groups consisted of 23 nonmusicians (12 males, mean age: 25.65 ± 3.42 y) and 48 musicians (26 males, mean age: 24.68 ± 4.39 y). The same procedure was followed to determine the relationship between WM abilities and brain activity underlying the recognition of musical sequences. This was performed independently for the musician and nonmusician groups.

## Supplementary Material

pgac216_Supplemental_Files

## Data Availability

The codes are available on GitHub (https://github.com/gemmaferu/MEG-auditory-memory/blob/main/papers/pnasnexus_associations_wm_recognition_auditory_sequences.m and https://github.com/leonardob92/LBPD-1.0.git). The anonymized neuroimaging data from the experiment are available on Zenodo (https://doi.org/10.5281/zenodo.7105001).
