# Supplementary material for: Associations between abstract working memory abilities and brain activity underlying long-term recognition of auditory sequences
Source: PNAS Nexus. 2022 Sep 28;1(4):pgac216. doi: 10.1093/pnasnexus/pgac216 (PMC9802106; doi:10.1093/pnasnexus/pgac216)
Supplement: pgac216_Supplemental_Files [file pgac216_supplemental_files.zip › PNASNEXUS-PNASNEXUS-2022-00572-s01.docx]

**Supplementary materials**

Supplementary materials related to this study are organized as supplementary figures and tables. Due to their large size, supplementary tables have been reported in Excel files that can be found at the following link:

<https://drive.google.com/drive/folders/1z3S7BTV7t5jfko6XDYprbdi84oJwgE9s?usp=sharing>


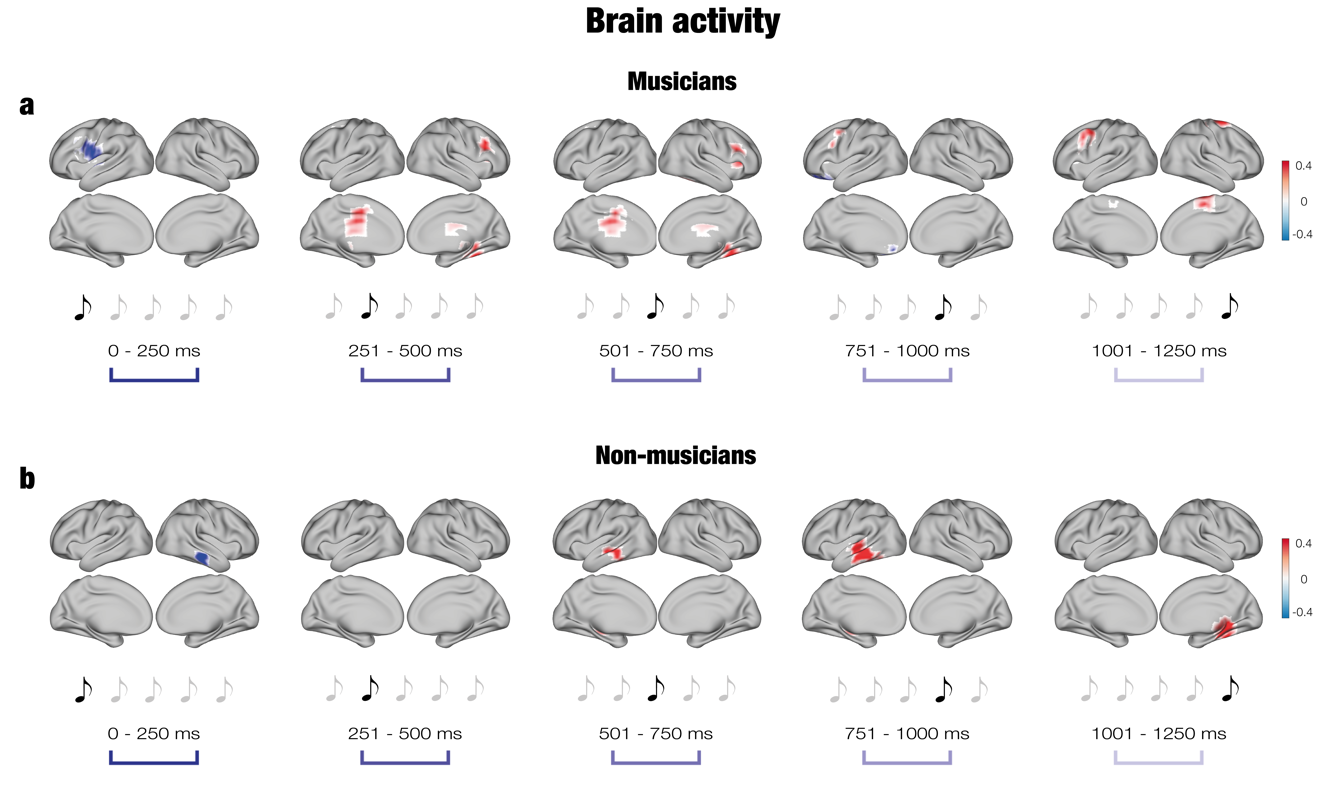


**Figure S1. Brain activity underlying the correlation between WM scores and recognition of auditory sequences in musicians and non-musicians.**

Significant Pearson’s correlations between the brain activity underlying recognition of the sequences and WM scores in musicians (**a**) and non-musicians (**b**). The correlations are depicted in brain templates in five subsequent time windows corresponding to the duration of each musical tone forming the sequences (as illustrated by the sketched musical tones above the time windows). The colorbar shows Pearson’s correlation coefficient obtained by correlating the brain activity underlying recognition of the previously memorized versus novel auditory sequences with the WM scores.

**Table S1. Significant clusters of activity for MEG source data (memorized versus novel sequences).**

Significant clusters of activity estimated from the contrasts between the brain activity (in 0.1 – 1 Hz) underlying memorized and novel musical sequences. The table depicts the contrast for each of the tones comprising the musical sequences, along with the brain regions, hemispheres, and *t*-values for each voxel.

**Table S2. Significant clusters emerged from the correlation between WM abilities and MEG brain data underlying recognition of previously memorized musical sequences.**

Significant clusters of activity estimated from the correlations between WM abilities and the brain activity (in 0.1 – 1 Hz) underlying previously memorized musical sequences. The table depicts the correlation for each of the tones comprising the musical sequences, along with the brain regions, hemispheres, and *r*-values for each voxel.

**Table S3. Significant clusters emerged from the correlation between WM abilities and MEG brain data underlying recognition of previously memorized musical sequences in the musician group.**

Significant clusters of activity estimated from the correlations between WM abilities and the brain activity (in 0.1 – 1 Hz) underlying previously memorized musical sequences in musicians. The table depicts the correlation for each of the tones comprising the musical sequences, along with the brain regions, hemispheres, and *r*-values for each voxel.

**Table S4. Significant clusters emerged from the correlation between WM abilities and MEG brain data underlying recognition of previously memorized musical sequences in the non-musician group.**

Significant clusters of activity estimated from the correlations between WM abilities and the brain activity (in 0.1 – 1 Hz) underlying previously memorized musical sequences in non-musicians. The table depicts the correlation for each of the tones comprising the musical sequences, along with the brain regions, hemispheres, and *r*-values for each voxel.
